# How Should Disaster Base Hospitals Prepare for Dialysis Therapy after Earthquakes? Introduction of Double Water Piping Circuits Provided by Well Water System

**DOI:** 10.1155/2016/9647156

**Published:** 2016-11-23

**Authors:** Naoki Ikegaya, George Seki, Nobutaka Ohta

**Affiliations:** ^1^Department of Medicine, Yaizu City Hospital, 1000 Dobara, Yaizu, Shizuoka 425-8505, Japan; ^2^Department of Nephrology, Yaizu City Hospital, 1000 Dobara, Yaizu, Shizuoka 425-8505, Japan; ^3^Department of Urology, Yaizu City Hospital, 1000 Dobara, Yaizu, Shizuoka 425-8505, Japan

## Abstract

After earthquakes, continuing dialysis for patients with ESRD and patients suffering from crush syndrome is the serious problem. In this paper, we analyzed the failure of the provision of dialysis services observed in recent disasters and discussed how to prepare for disasters to continue dialysis therapy. Japan has frequently experienced devastating earthquakes. A lot of dialysis centers could not continue dialysis treatment owing to damage caused by these earthquakes. The survey by Japanese Society for Dialysis Treatment (JSDT) after the Great East Japan Earthquake in 2011 showed that failure of lifelines such as electric power and water supply was the leading cause of the malfunction of dialysis treatment. Our hospital is located in Shizuoka Prefecture, where one of the biggest earthquakes is predicted to occur in the near future. In addition to reconstructing earthquake-resistant buildings and facilities, we therefore have adopted double electric and water lifelines by introducing emergency generators and well water supply systems. It is very important to inform politicians, bureaucrats, and local water departments that dialysis treatment, a life sustaining therapy for patients with end stage renal diseases, requires a large amount of water. We cannot prevent an earthquake but can curb the extent of a disaster by preparing for earthquakes.

## 1. Introduction

Mass natural disasters cause widespread and severe damage on the delivery of dialysis services. Disasters such as earthquakes may result in two nephrologic patient populations to manage those with crush syndrome and AKI and those already undergoing chronic dialysis. On August 29, 2005, Hurricane Katrina resulted in the worst urban disaster in modern American history. Katrina forced the closure of 94 dialysis facilities in New Orleans and the Gulf region, largely due to loss of electrical power or flooding [1]. Nearly 6,000 dialysis patients were affected, and most missed dialysis care and had to find another dialysis clinics [2, 3]. The event highlighted the vulnerability of the poor, the elderly, and patients with chronic diseases, and especially of patients with end stage renal disease (ESRD).

In addition, major earthquakes are followed by a considerable numbers of patients with crush syndrome. The incidence of crush syndrome has been assessed at 2 to 5% at least. Approximately 50% of the patients with crush syndrome develop AKI, and approximately 50% of those with AKI will need dialysis. In the 1999 Marmara earthquake, 477 patients with crush syndrome needed dialysis therapy [4]. In the 1995 Hanshin-Awaji earthquake in Japan, 123 patients with crush syndrome were dialyzed [5, 6].

On the other hand, disasters result in the damage of infrastructure such as medical facilities, lifelines, and transportation routes. The ability to provide hemodialysis to chronic dialysis patients and patients with AKI is impacted [7–10].

In April 2016, this year, a series of earthquakes struck Kumamoto Prefecture in Japan's Kyusyu Region and dozens of people died. Twenty-two out of 94 dialysis facilities in Kumamoto Prefecture could not continue dialysis therapy because of damage in medical infrastructures [11]. From now on, the precise data about the damage caused by these earthquakes will be announced. Japan, located in an area with frequent earthquakes, has experienced devastating earthquakes in the past. The aim of this article is to review the damage in dialysis facilities caused by the recent earthquakes in Japan, where hemodialysis (HD) is much more popular than peritoneal dialysis [12]. In addition, we wish to emphasize the importance of preparedness for earthquakes by introducing well water supply system.

## 2. Previous Earthquakes in Japan and Dialysis:* The Great Hanshin Earthquake and Dialysis*

In January 1995, the Hanshin-Awaji Earthquake, with a magnitude of 7.2 on the Richter scale, struck the Hanshin area around the city of Kobe, where the social and economic functions are concentrated, and killed more than 6,000 people. About 50 out of 104 dialysis facilities were affected [17]. Of all the dialysis centers in the affected areas, two were completely destroyed, and 28 lightly damaged. Only two centers escaped destruction. Public facilities, such as water pipes, communication lines, and gas pipes, were damaged. Electric power failures occurred all over the city, but 80% of the affected areas recovered within 24 h and within 5 days the problem was solved. Every HD center or hospital was provided with a water tank, which has a capacity to meet about half the normal daily consumptions. During the earthquake, more than 20% of centers lost the major part of stored water. Restoring water supply and drainage required 12.8 days on average, the maximum being 37 days. Forty-three hospitals received water wagon services. It was not sufficient so that 15 hospitals had to keep water by their own efforts. At that time, the local government and the local water authority did not fully recognize the necessity of such a large quantity of water for dialysis treatment, a life sustaining therapy for patients with end stage renal diseases. Since then, many nephrologists who have experienced this disaster at dialysis centers thought it very important that the local government and water department recognize and share the idea that dialysis treatment is a life sustaining therapy requiring about 120 L of pure water for a single standard dialysis. After The Great Hanshin-Awaji earthquake, the Ministry of Health, Labour and Welfare (MHLW) instituted the disaster base hospital in 1996. The disaster base hospital is expected to play a key role in the most acute phase of a large-scale disaster. The requirement criteria as a disaster hospital by MHLW are described as follows [18].

*Disaster Base Hospital Designation Requirements [18]*

1. Accepting all seriously injured or ill patients from the stricken area around the clock
2. Conducting the aeromedical shuttling by helicopter for patients and medical supplies between the disaster base hospital in the stricken area and disaster base hospital outside the stricken area
3. Holding disaster dispatch medical care team (DMAT (disaster medical assistance team))
4. Having surge capacity (two times for inpatients and five times for outpatients)
5. Earthquake-resistant structure
6. In-hospital generator, capable of operation 60% of the hospital's electrical needs, and with fuel for three days
7. Tray water tank of appropriate capacity and possession of the well
8. Helicopter landing pad at the hospital site
9. Having the following practice equipment:
  - Satellite phone
  - Satellite line Internet
  - Multiple means of communication
  - Emergency Medical Information System (EMIS)
  - Lifesaving medical care kits for the seriously ill emergency patients
  - Carrying-type lifesaving medical care equipment, medical supplies, tent, generator, drinking water, food, life supply, and triage tag
  - Emergency vehicle or ambulance

By April 2016, 712 hospitals including ours had been designated as disaster base hospitals [19].

## 3. Previous Earthquakes in Japan and Dialysis:* The Great East Japan Earthquake and Dialysis*

On March 11, 2011, the Great East Japan Earthquake triggered a massive tsunami along the Pacific Coast of northeastern Japan and killed nearly 20,000 people. The survey performed by Japanese Society for Dialysis Therapy (JSDT) showed that 315 out of 3886 dialysis facilities in the Eastern Japan were nonfunctional by damage caused by the earthquake [20]. They identified eight causes for HD center failure at that time: (1) structural damage to buildings by earthquake, (2) damage to dialysis equipment by earthquake, (3) failure of the electrical power supply, (4) damage by tsunami, (5) damage by nuclear power plant accident, (6) interruption of the water supply, (7) shortage of dialysis materials, and (8) lack of dialysis workforce. They showed that the leading two causes for long-term (more than 3 days) malfunction were interruption of water supply and damage to buildings. The survey concluded that serious effects on HD centers were classified into three types of failures: (1) failure of lifelines such as electric power and water supply (80%), (2) damage to buildings (15%), and (3) damage by special causes such as tsunami, nuclear power plant accident, and shortage of supply such as dialysis material and fuel forth (5%). Hence HD centers must prepare for disasters in terms of these three factors.

## 4. Damage in Water Supply System in the Great East Japan Earthquake

Matsumura et al. reported the damage to water supply facilities and state of water resource operation at disaster base hospitals in Miyagi Prefecture (Japan) in the wake of the Great East Japan Earthquake [21]. Nine out the 14 hospitals experienced cuts to their water supplies, with a median value of three days (ranging from one to 20 days). Three out of 4 hospitals, which had well water supply facilities, were able to obtain water from their wells even after the disaster. The volume of water from these wells was quite comparable to that supplied under normal operating conditions. The survey proved that well facilities that can produce pure water are very effective in the event of an emergency. They concluded that it is possible to minimize the disruption or reduction of hospital functions in the event of a disaster through the proper maintenance of water facilities by securing alternative sources of water, such as well water. At the same time, it is desirable to conclude water supply agreements and to formulate water allocation plans. However, frequent personnel shifts in Japanese local governments sometimes make it difficult to continue such agreements or plans. For this reason, several dialysis centers in Fukushima Prefecture, after experiencing the difficulty in getting water for dialysis therapy at the Great East Japan Earthquake, proposed a bill to the municipal council to secure sufficient water for dialysis treatment from the local government with priority at a disaster [22].

## 5. Future Earthquakes

Shizuoka Prefecture, where our hospital is situated, is considered to have one of the biggest earthquakes in the near future. In 1976, Mr. Katsuhiko Ishibashi, an assistant at the Faculty of Science, University of Tokyo, theorized: “It would not be surprising if a large-scale earthquake occurred in the Tokai area centering around Shizuoka Prefecture tomorrow [23].” After a serious social problem brought about by the publication of this earthquake theory, it has become the most urgent task for the prefecture, cities, and families to prepare for a Tokai Earthquake. Over forty years have passed without a major earthquake in this area since this theory was published, but seismologists are in agreement with each other that “the occurrence of a Tokai Earthquake is more likely day by day.” The recent data suggest that a megathrust earthquake is predicted to occur in not only Tokai but also Nankai area almost at the same time [15]. The local government reconstructed earthquake-resistant hospitals, schools, and social welfare facilities. Japanese Ministry of Health, Labour and Welfare reported in 2015 that the rate of earthquake-resistant buildings in disaster base hospitals is 95.5% in Shizuoka Prefecture, highest in Japan, while it is 85.2% in Japan, on average.

## 6. How Should We Prepare for Earthquakes?

**Figure 1:**
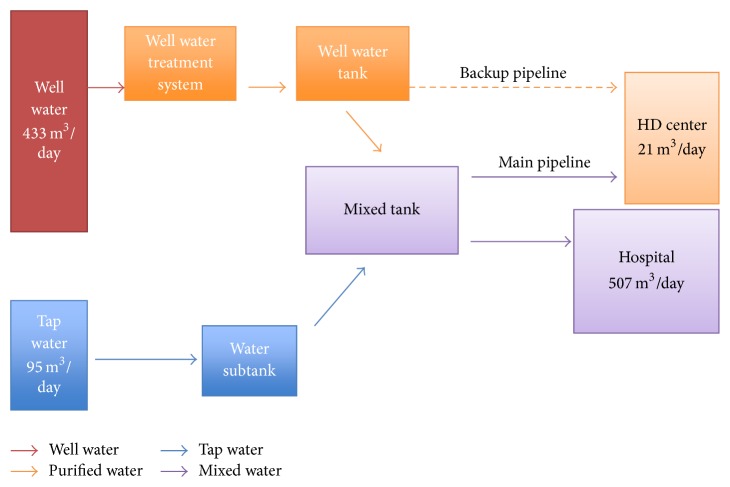
Water treatment system in Yaizu City Hospital. Double water pipelines for the HD center have been installed in our hospital.

Above results have suggested a means by which HD center of disaster base hospitals can prepare for earthquakes. In addition to earthquake-resistant buildings and facilities, it is essential to secure water supply systems. Since our hospital is a disaster base hospital and maintained by the city, the director of our hospital regularly meets the mayor and governmental officials and informs them of the necessity of a large amount of water for hospital functions. Now our city bureaucrats well recognize the requirement of water supply for maintaining hospital roles. In addition to securing water allocation plans by the city, a well water facility and double water piping circuits have been installed in our hospital (Figure 1) [13]. Our hospital, having 471 inpatient beds and 35 HD beds, usually consumes about 500 m^3^ of water daily. This well water system equipped with a reverse-osmosis purification devise can supply ultrapure water in a volume corresponding to 85% of daily water consumption in our hospital. Moreover, emergency electric power supply systems have been employed, making our lifelines even more stable.

## 7. Discussion

In this paper, we presented damage of dialysis facilities observed in recent earthquakes. Earthquakes resulted in the damage of infrastructure such as buildings, lifelines, and transportation routes. The leading two causes for the long-term malfunction of dialysis facilities were interruption of water supply and damage to buildings. Hence we reconstructed earthquake-resistant buildings and facilities, and we have adopted double water lifelines by introducing well water supply systems in our hospital. In addition, the director of our hospital regularly meets the officials of the water department in the city and stresses that a large amount of pure water is vital in the dialysis treatment.

In 2005, Hurricane Katrina disclosed serious problems in preparedness for dialysis patients, facilities, and ESRD networks. In 2006, The Kidney Community Emergency Response (KCER) Coalition was organized in 2006 [14]. The mission of KCER is to collaboratively develop, disseminate, implement, and maintain a coordinated preparedness and response framework for the kidney community. KCER provides resources specifically for dialysis facilities to help them prepare, respond, and recover from disasters. One of the resources for dialysis facilities is “Disaster Preparedness: A Guide for Chronic Dialysis Facilities” in their HP. The guide describes that dialysis units with access to on-site water via a well were able to function during the Hurricane Katrina and it is necessary to establish a good working relationship with the municipal water supplier, educate them about the importance of water to your patients, and teach them about your water treatment system and its limitations. There are several resources to guide planning preparation for a disaster [14, 16, 24–26] and a list of online resources are shown in Table 1.

Irrespective of the event of Hurricane Katrina, most dialysis patients in North Carolina were unprepared for a disaster. Dialysis providers should educate dialysis patients and rehearse a disaster plan with patients regularly [26].

## 8. Implications for Practice

Dialysis providers should be aware that a disaster results in the failure of the provision of hemodialysis services. In addition, patients with crush syndrome and AKI may appear after major earthquakes. The leading two causes for the long-term malfunction of dialysis facilities were interruption of water supply and damage to buildings. It is necessary for dialysis providers to prepare in advance for a disaster using online resources such as KCER.

## 9. Conclusions

An earthquake is a geological event that is beyond human control. But we can at least curb the extent of a disaster. Now is the time to draw lessons from malfunction in HD treatment we have experienced during the previous large earthquakes.

## Figures and Tables

**Table 1:** Online resources for dialysis units and patients.

Organization	Contact	Target audience	Resources
Centers for Disease Control and Prevention	http://www.cdc.gov/disasters/dialysis.html	Dialysis providers	Dialysis Care After a Disaster [13]
National Kidney Foundation	https://www.kidney.org	Patients	Planning for Emergencies [14]
Kidney Community Emergency Response (KCER) Program	http://www.kcercoalition.com	Dialysis providers	Disaster Preparedness: A Guide for Chronic Dialysis Facilities [15]
The ISN Renal Disaster Relief Task Force (RDRTF)	http://www.theisn.org	Dialysis providers	Renal Disaster Relief [16]
